# Association of ovarian teratoma with anti-N-methyl-D-aspartate receptor encephalitis: a case report and narrative review

**DOI:** 10.1007/s00404-024-07779-6

**Published:** 2024-11-04

**Authors:** Konrad Joseph, Sarah van der Hock, Ishith Seth, Nipuni Hapangama, Lara Gibson, Roberto Cuomo, Warren M. Rozen, Nita Dhupar

**Affiliations:** 1 Department of Surgery, Port Macquarie Hospital, Port Macquarie, NSW Australia; 2 Faculty of Science, Medicine, and Health, The University of Melbourne, Melbourne, VIC Australia; 3 Faculty of Science, Medicine, and Health, Monash University, Melbourne, VIC Australia; 4 Department of Obstetrics and Gynaecology, Murrumbidgee Health, Wagga Wagga, NSW Australia; 5 Department of Obstetrics and Gynaecology, Joan Kirner Women’s and Children’s Hospital, Western Health, St Albans, Australia; 6 Department of Medicine, Surgery and Neuroscience, University of Siena, Siena, Italy

**Keywords:** Ovarian teratoma, Encephalitis, NMDA, Receptor

## Abstract

**Background:**

Anti-N-methyl-D-aspartate receptor (NMDAR) encephalitis is a potentially life-threatening autoimmune disorder which is strongly associated with ovarian teratomas in young female patients. The primary aim is to highlight the importance of considering NMDAR encephalitis in the differential diagnosis of young female patients presenting with acute or subacute neuropsychiatric symptoms, especially when accompanied by ovarian teratomas.

**Case description:**

This case report and literature review detail the presentation, diagnosis, and treatment of a 35-year-old G4P3 Indigenous woman who initially presented with neuropsychiatric symptoms and fever, having a history of extensive drug and alcohol use. Misdiagnosed initially, the patient's lack of response to standard treatments led to further investigations, revealing paraneoplastic anti-NMDAR encephalitis secondary to a left ovarian teratoma. The report examines the treatment regimen followed, including prednisolone, intravenous immunoglobulin, rituximab injections, and laparoscopic bilateral salpingo-oophorectomy.

**Conclusions:**

This case underscores the critical need for increased clinical vigilance for anti-NMDAR encephalitis in patients, particularly young females, presenting with neuropsychiatric symptoms and potential ovarian teratomas. The literature review accompanying the case report provides valuable insights into the presentation, diagnosis, and management of this complex condition. Lastly, this study emphasised the diagnostic challenges inherent in paraneoplastic neuropsychiatric syndromes, advocating for a multidisciplinary approach in similar clinical scenarios.

## Introduction

Anti-N-methyl-D-aspartate receptor encephalitis (anti-NMDARE) is a severe but treatable autoimmune disorder first described in 2007 by Dalmau and colleagues [1, 2]. Along with awareness and testing, the incidence of this condition has since increased worldwide [3]. It has various clinical manifestations, with most patients experiencing a nonspecific prodromal phase suggestive of a viral flu-like illness before the emergence of psychiatric symptoms that may mimic that of schizophrenia or substance-induced psychosis [4, 5]. Neuropsychiatric manifestations may involve auditory or visual hallucinations, delusions, agitation, catatonia, mood disturbances, anxiety, and anorexia. The onset is notably rapid, within one to three weeks, and often presents in patients with no prior psychiatric history [2]. Differentials for this constellation of symptoms may include primary psychiatric disorders, drug misuse, neuroleptic malignant syndrome, migraines, effects of antipsychotic medications, and infectious encephalitis [4]. Whilst carefully excluding these conditions, clinicians should exhibit a high clinical suspicion for anti-NMDARE. Diagnosis is achieved primarily through cerebrospinal fluid (CSF) assays for the presence of immunoglobulin G (IgG) anti-NMDAR antibodies [4]. The variability in presentation and potential for misdiagnosis of this condition highlights a need for further research to improve clinical recognition and improve patient outcomes.

It has been established that anti-NMDARE predominantly affects young adult women [4, 6]. Paraneoplastic associations have also been demonstrated, particularly with ovarian teratoma which is a common form of ovarian germ cell tumour [4, 6, 7]. This case report describes a recent case of anti-NMDARE associated with ovarian teratoma, adding to the existing literature, and furthering our understanding of this disease relationship. It is imperative to appreciate the association between anti-NMDARE and ovarian teratoma, which requires different management to anti-NMDARE in isolation for resolution and good long-term prognosis [8]. The following literature review consolidates the most recent information on teratoma-associated anti-NMDARE, summarises relevant management guidelines and compares our case with other descriptions.

## Case report

A 35-year-old G4P3 woman of Indigenous background was brought in by ambulance to the emergency department after displaying erratic and aggressive behaviour at home. Her partner reported behaviours including running outside at night, knocking on neighbours’ doors, and smashing windows. She was experiencing visual hallucinations and suicidal ideation. Upon arrival at the hospital, the patient was managed through verbal de-escalation and administration of 10 mg IM of droperidol and placed as an involuntary admission under the Mental Health Act (2007).

The patient was unable to provide a coherent history at the time, though mentioned precipitating factors for her distress included the deaths of several close family members. Collateral history confirmed that the patient used cannabis and alcohol regularly, but her partner denied her having ingested alcohol or cannabis for the preceding days. She had also experienced 10 days of cold and flu-like symptoms with light-headedness and back pain that was managed with over-the-counter medication. Her past medical history included depression with a desvenlafaxine overdose in 2017 and well-controlled asthma. The patient’s documented obstetric history included uncomplicated vaginal deliveries of her three children and a long-term Implanon in place since.

On examination, the patient was febrile to 38.3 degrees, but all other vitals were within normal limits. A mental state exam revealed speech was circumstantial and confused, with a reactive mood and labile affect. She appeared to be reacting to visual stimuli at the time of assessment. She denied any thoughts of self-harm or suicidal ideation and her overall insight and judgement was poor. A full blood count, electrolytes and inflammatory markers were requested, which were largely unremarkable except for mild hyponatremia (127) and hypokalaemia (2.5 mmol) which was attributed to alcohol use. Her urine MCS revealed the presence of *E.coli* and a CT brain revealed a small midline frontal scalp subgaleal haematoma which was sustained during her period of erratic behaviour at home.

The patient at this time was concluded to have withdrawal delirium secondary to her alcohol and cannabis intake. She was treated with Augmentin Duo Forte for her UTI and benzodiazepines, thiamine, fluid and electrolyte replacement for withdrawal management. Her aggression continued despite treatment and so quetiapine, an anti-psychotic, and sodium valproate, a mood stabiliser, were added after which she was intubated in ICU. After discussions with the neurology team, she was transferred to a tertiary centre four days later due to concerns about a differing cause of her symptoms.

On examination, further neurological exam findings included slight vertical nystagmus and eyelid flickering, with myoclonus of the lips and tongue and increased upper limb tone. The patient was subsequently investigated for infective or autoimmune encephalitis. An MRI of the brain showed mild dural enhancement. Her EEG demonstrated diffuse cortical slowing. Preliminary CSF investigations revealed elevated protein (2.16 g/L) and elevated neopterin (302.34 nmol/L). On day 8 of admission, anti-NMDA receptor antibodies were detected in the cerebrospinal fluid. Albumin EPG was low at 32 g/L, alpha-1globulin elevated to 2.5 g/L, and oligoclonal bands were prominent in CSF, supportive of CNS inflammation. She was subsequently commenced on prednisolone and intravenous immunoglobulin as a treatment for her autoimmune encephalitis. A 6-monthly rituximab injection was prescribed following concerns for neurological deterioration with vertical nystagmus and apnoeic episodes.

**Fig. 1:**
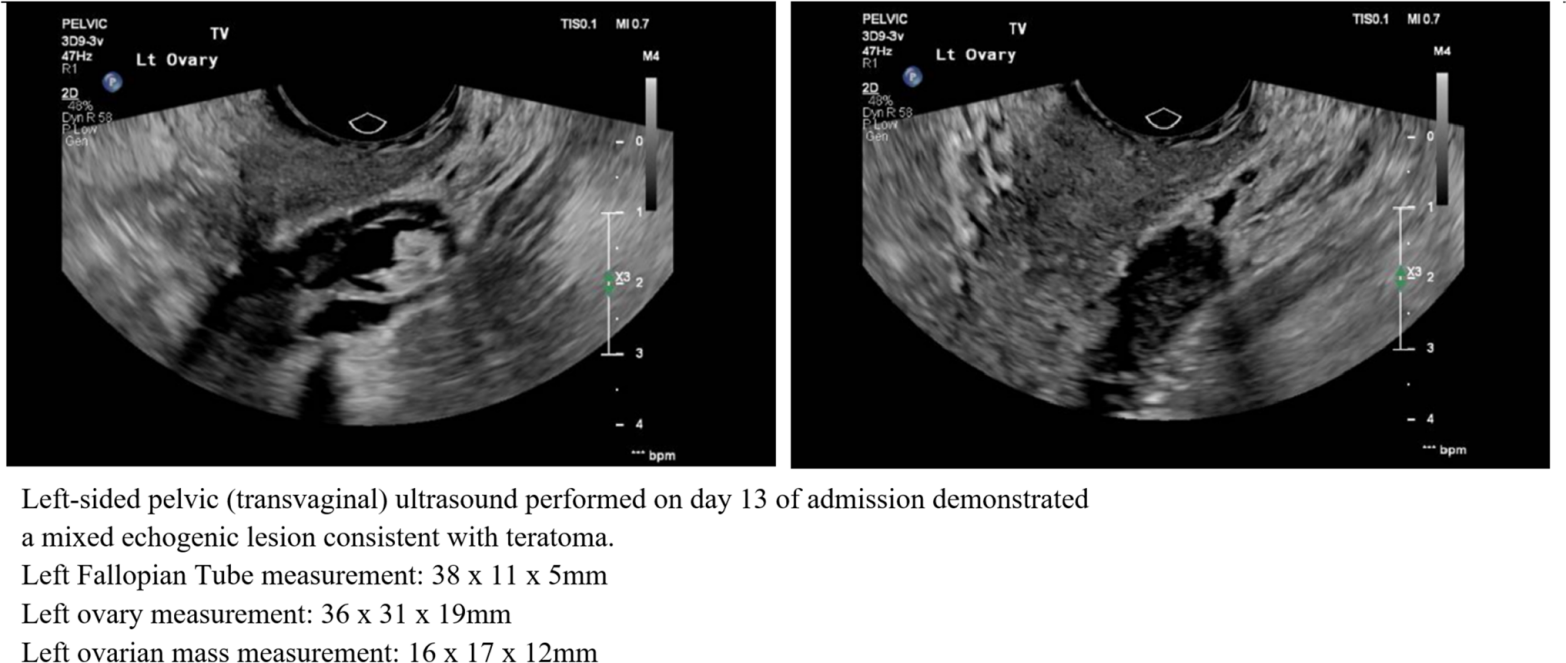
Left-sided pelvic ultrasound

An underlying ovarian aetiology was suspected given the association between gynaecological cancers and autoimmune encephalitis. Lab investigations for serum tumour markers CEA, Ca19-9 and Ca-125 were unrevealing. On day 13 of admission, a mixed echogenic lesion (dimensions 16 × 17 x 12 mm) consistent with teratoma was detected via transvaginal ultrasound on the left ovary (Fig. 1). To minimise the risk of spillage, and after discussion regarding implications for future fertility and risk of surgical menopause with the patient’s family and the hospital guardianship board, it was agreed that laparoscopic bilateral salpingo-oophorectomy would be conducted. The procedure was uncomplicated. Gross inspection of the mass in question revealed skin, hair and fat, and subsequent histopathology confirmed a mature cystic teratoma of the left ovary. The histologic appearance of the tissue was consistent with that of mature cystic teratoma. Samples revealed keratinising squamous epithelium with adnexal structures, neural tissue, mucinous glands, fat cells and respiratory epithelium. A long-stalked simple cyst (dimensions 15 × 5 x 3 mm) was attached to the right ovary. Fallopian tubes had normal macro- and microscopic structures bilaterally.

The patient has continued to reside within the hospital for a collective total of 13 months due to ongoing fluctuating aggression and sexually inappropriate behaviours. Her stay has been complicated by multiple hospital-acquired infections, deep vein thromboses, and an osteoporotic hip fracture after a fall, all of which have been treated medically. An investigation into the cause of osteoporosis at the patient’s relatively young age was undertaken, in which poor diet was revealed to be a key risk factor. Discussions regarding weaning medications, discharge planning and support for the family are continuing (Table 1).

**Table 1:** Clinical Timeline to Surgical Intervention

Day	Clinical information
(− 10)	Onset of prodromal symptoms
(− 1)	Onset of neuropsychiatric symptoms
0	Presentation to hospital
	Initial blood labs in ED were unremarkable
	Small subgaleal hematoma identified on CT Brain
	Diagnosis of delirium secondary to alcohol and UTI
	Initiation of antibiotics, benzodiazepines, thiamine, fluid and electrolyte replacement
0	Admission to ICU
0–4	Continued neuropsychiatric symptoms—intubated on Day 4 due to escalating sedation requirements
4	Transfer to a tertiary care centre
8	CSF studies
	Diagnosis of anti-NMDARE
	Initiation of immunotherapy
13	Trans-vaginal ultrasound investigation
	Diagnosis of mature ovarian teratoma
15	Bilateral salpingo-oophorectomy

## Methodology

Two independent researchers (Author 1 and Author 3) performed a literature search from January 1901 to June 2023 across various academic databases including PubMed, Cochrane Library, and Embase and others using specific search terms comprising "NMDA-receptor antibodies encephalitis”, “ovarian teratoma”, “autoimmune encephalitis”, “NMDA receptor", “paraneoplastic neurological syndrome[s]” and "neuropsychiatric disorder[s]". Titles and abstracts were screened for relevance and any discrepancies resolved through discussion or third-party consultation. The data extracted from the articles will be narratively synthesised to provide a holistic overview of the condition.

## Literature review

The paraneoplastic association between anti-NMDARE and ovarian teratoma has been recognised since initial descriptions by Dalmau and colleagues in 2007, with an underlying germ-cell tumour (GCT) found in a large percentage of patients [1, 9]. In 2021, Wu and colleagues reviewed 1058 cases of anti-NMDARE and found that 37.4% of women who experience this condition have an underlying GCT [7]. The incidence of an underlying tumour in patients with anti-NMDARE is highest in black and Asian women aged 12 to 44, and the most common GCT associated with anti-NMDAR is by far ovarian teratoma [7]. Ovarian teratomas may be classified as mature (benign), or immature (malignant) [7]. A unilateral mature ovarian teratoma is the most likely subtype to be present in the context of anti-NMDAR; it tends to be smaller, is associated with older patient age, and is associated with better outcomes when compared with immature teratoma [7, 10].

It is believed that teratomas with neural tissues could trigger an immune response, resulting in the overproduction of anti-NMDAR antibodies, reacting in the hippocampus and forebrain regions, leading to an immune-mediated response causing the complex neuropsychiatric syndrome [7, 13]. Most commonly seen in ovarian teratoma, the autoantibody NMDAR-IgG has direct pathogenic potential on the target GluN1 antigen [7]. In 2022, Li and colleagues found 3.07% of patients with histopathology confirmed ovarian teratomas were diagnosed with NMDARE [28].

Despite the variability of presentation, prior reports support the notion that anti-NMDARE often undergoes predictable clinical phases [11–13]. The first discrete stage is the viral prodrome which is experienced by 70% of patients, lasting an average of 5 days [14]. Initial psychiatric changes often follow, lasting between one to three weeks. Symptoms experienced during this stage are broad and varied. Consequently, patients are often misdiagnosed with a primary psychotic or substance-induced disorder during this phase. After several weeks, patients enter the next discrete stage characterised by neurological complications such as autonomic instability, abnormal movements, and seizures. Patients are often treated in ICU during this stage and experience intermittent neurological symptoms amid fluctuating levels of consciousness. Upon successful recognition of anti-NMDARE and initiation of treatment, patients enter their recovery phase, requiring admission for up to four months if not longer. With early detection and removal of the teratoma, most patients will achieve a favourable long-term prognosis [8].

Diagnostic criteria were published in 2016 by Graus and colleagues to aid in the recognition of probable anti-NMDARE [15]. According to these guidelines, a diagnosis can be made when all three of the following criteria are met:

1. Subacute onset (less than three months) of working memory deficits, altered mental status, or psychiatric symptoms
2. At least one of the following:

o New focal CNS findings
o Seizures not explained by a previously known seizure disorder
o CSF pleocytosis
o MRI features suggestive of encephalitis
o Abnormal EEG

(3) Reasonable exclusion of alternate causes

*Diagnosis can also be made in the presence of three of the above groups of symptoms accompanied by a systemic teratoma

Early suspicion is important as the absence of appropriate treatment can render the condition fatal [9]. Before reaching a diagnosis and starting therapy, it is important to rule out other conditions such as schizophrenia and drug-induced psychosis.

If anti-NMDARE is suspected, CSF studies should be obtained [15]. CSF protein may be elevated, revealing lymphocytic pleocytosis or oligoclonal bands. IgG antibodies against the NR1 subunit of the NMDAR in CSF are the primary indicator of this disorder [6]. In their 2022 observational study on 192 patients with anti-NMDARE, Zhang and colleagues found that patients with teratoma tend to have a higher CSF IgG titre than those without [8]. Previous studies have found that laboratory testing for serum anti-NMDAR antibodies is less sensitive than CSF analysis [16]. However, these tests are often pursued simultaneously.

Electroencephalography (EEG) can also provide valuable information. In 2018, van Sonderen and colleagues published an article exploring the predictive value of EEG in anti-NMDARE finding that 90% of patients with anti-NMDARE show EEG abnormalities [17]. EEG tracings in anti-NMDARE are characterised by focal generalised slow delta activity with or without superimposed high frequency discharges [18].

Combination immunotherapy involving steroids, intravenous immunoglobulins and plasma exchange is crucial, and second-line immunotherapy involving cyclophosphamide and rituximab should be carried out promptly for non-responders [3]. ICU support may be required for some patients, with 20% of patients requiring intubation secondary to central hypoventilation [19]. Hospitalisation durations of up to four months are typical [14]. The mortality rate of anti-NMDARE is about 6%, with approximately 50% of patients achieving full recovery [6]. Early treatment is associated with quicker recovery and decreased risk of relapse [9, 20].

It is an important consideration that patients with teratoma generally have a poor response to immunotherapy alone and require concomitant surgical resection of the mass [8]. Mature teratoma is usually removed with simple excision whereas immature teratoma is removed via bilateral salpingo-oophorectomy [21]. Recovery rates are higher in women with mature teratoma (88%) than immature teratoma (76%) [21]. In refractory cases where imaging is negative and antibodies are positive, an exploratory laparotomy may be considered to rule out microscopic teratoma, although this issue remains controversial [22]. Due to the rarity of the condition, screening for anti-NMDAR antibodies in patients with ovarian teratoma is not indicated currently [23].

## Case discussion

Our patient fits the typical demographic for this condition of being a young female adult. Her initial presentation to a healthcare service occurred during the neuropsychiatric phase of her illness. This along with her subsequent misdiagnosis of alcohol-induced delirium is also consistent with previous cases of anti-NMDARE[4]. The initial diagnosis for our case was complicated by the patient’s comorbid conditions and patient background (concomitant UTI, drug and alcohol history). In addition to following the classic clinical phases of anti-NMDARE, the results of diagnostic tests were consistent with published criteria and previous literature. Antibody positivity and pleocytosis were noted in CSF studies, as were EEG abnormalities.

The time to diagnosis and subsequent tumour resection in our case was much faster than typical. The median date from onset to ovarian teratoma diagnosis is 22.5 days [24]. Our patient’s swift treatment within 15 days despite initial misdiagnosis was due to recognition of this disease association by our team, showcasing the importance of clinician awareness of this condition.

The size of this patient’s teratoma was small at 16 × 17 × 12 mm, consistent with other case reports [25]. It should also be noted that although ovarian teratoma is typically removed with simple excision, our team decided to take a conservative approach and perform a laparoscopic bilateral salpingo-oophorectomy to minimise the risk of content spillage and recurrence [26]. Recurrence of dermoid cysts and associated encephalitis has been reported after unilateral surgery [27]. This option was chosen after a discussion of the case within the team, with the guardianship committee, and with the patient and her family members. As with many patients who experience anti-NMDARE, our patient required ICU support and is yet to experience full resolution of her symptoms nearly 13 months on. She continues to progress through a prolonged recovery phase and the lasting impacts of her condition are yet to be fully seen.

Further research exploring the molecular subtypes and immunoreactivity of ovarian teratomas will allow us to better understand their associations with anti-NMDARE. Official guidelines for the specific diagnosis and management of teratoma-associated anti-NMDARE should be developed given the high proportion of women with anti-NMDARE who have an underlying GCT [7].

## Conclusion

This case study and literature review present the rare yet clinically significant phenomenon of teratoma-associated anti-NMDARE in a 35-year-old female patient presenting with neuropsychiatric symptoms and fever. This paper reinforces our evolving understanding of the teratoma-associated anti-NMDARE, consolidates current management guidelines and suggests areas for future exploration.

## Clinical messages

The severity and treatability of this syndrome underscore the need for its inclusion in the differential diagnosis of patients presenting with neuropsychiatric symptoms not improving with standard treatment.

We emphasise the importance of early recognition of teratoma-associated anti-NMDARE based on an understanding of its characteristic clinical phases and epidemiologic factors such as gender, age, and ethnicity.

Early surgery and immunotherapy are imperative for optimising patient outcomes. An individualised approach should be taken with consideration of the psychosocial implications on patient fertility and wellbeing.

## Data Availability

Data available on request from the authors due to patient confidentiality/privacy.
